# Effect of Different Groundwater Levels on Seismic Dynamic Response and Failure Mode of Sandy Slope

**DOI:** 10.1371/journal.pone.0142268

**Published:** 2015-11-11

**Authors:** Shuai Huang, Yuejun Lv, Yanju Peng, Lifang Zhang, Liwei Xiu

**Affiliations:** Institute of Crustal Dynamics, China Earthquake Administration, Beijing, 100085, China; Beihang University, CHINA

## Abstract

Heavy seismic damage tends to occur in slopes when groundwater is present. The main objectives of this paper are to determine the dynamic response and failure mode of sandy slope subjected simultaneously to seismic forces and variable groundwater conditions. This paper applies the finite element method, which is a fast and efficient design tool in modern engineering analysis, to evaluate dynamic response of the slope subjected simultaneously to seismic forces and variable groundwater conditions. Shaking table test is conducted to analyze the failure mode and verify the accuracy of the finite element method results. The research results show that dynamic response values of the slope have different variation rules under near and far field earthquakes. And the damage location and pattern of the slope are different in varying groundwater conditions. The destruction starts at the top of the slope when the slope is in no groundwater, which shows that the slope appears obvious whipping effect under the earthquake. The destruction starts at the toe of the slope when the slope is in the high groundwater levels. Meanwhile, the top of the slope shows obvious seismic subsidence phenomenon after earthquake. Furthermore, the existence of the groundwater has a certain effect of damping.

## Introduction

The stability of the slope is one of the leading problems in practical engineering, and the slope related geo-hazards pose a great threat to human life, infrastructure and properties all over the world [1],[2],[3]. During the recent earthquakes, it was presumed that the slope failures were caused mainly by high ground water levels in the slopes. Earthquake and groundwater are the common reasons of slope instability. Thus, it has received increasing attentions and become a hot topic in recent years[4],[5],[6],[7]. However, the dynamic response and failure mode of the slope subjected simultaneously to seismic forces and variable groundwater conditions still remain a difficult problem.

The failure of the slope under earthquake or groundwater is a crucial issue for the slope stability [8],[9],[10]. Unfortunately, the role of the mechanics in which earthquake loading and groundwater affect simultaneously the slope stability has not been addressed sufficiently. Most studies concerning earthquake or groundwater induced slope failure have ignored the dynamic coupling of seismic load and groundwater. Cai et al. [11] studied the influence laws of ground water levels on slope seismic stability and deformation based on pseudo static method in which the seismic load is considered as pseudo static, and found that the sliding scale of the slope will increase in groundwater. However, they ignored the dynamic pore water pressure when they considered the influence laws of ground water levels on slope seismic stability and deformation. Lu et al. [12] have evaluated the seismic slope stability of hypothetical and actual slopes affected by high ground water levels using the time history method. They find that the slope is easier to be damaged by the earthquake when the slope is in the groundwater; while the cumulative plastic displacement of the slope which is in the groundwater, as compared to the slope where there is no groundwater, is larger. Although the earthquake is considered as the dynamic load, one major problem for the research is that the groundwater is considered as the reduction of shear strength of the slope. Bi [13], Srilatha [14] and Kokusho [15] have also studied the dynamic response of the slope under earthquake, and found that accelerations are much amplified at the top of the slope. Furthermore, Viratjandr [16], Wang [17] and Ng [18] have evaluated the influence laws of the groundwater levels on stability of the slope, in which they find that the stability of slopes is been seriously influenced by the groundwater. Lin et al. [19], [20], [21], [22] performed a shaking table model test to reveal the dynamic behavior of a railway embankment slope, in which they find that the embankment slope exhibited a significant amplification effect on the input acceleration, and the acceleration response differs greatly when the slope subjected to different seismic excitations. Obviously, these methods consider only the seismic load or groundwater factor when they evaluated the slope stability under earthquake and completely neglected the dynamic pore water pressure, which is a main factor affecting the slope stability under earthquake.

Despite many researches have attempted to understand the mechanisms of slope stability and model slope stability which is in various conditions, it is difficult to realistically evaluate slope stability considering the dynamic coupling of seismic load and groundwater. In practical designs, pseudo-static method is usually used to evaluate the safety of a slope. However, this method could not consider the influence of strong motion duration and dynamic amplification of the earthquakes. In fact, the research methods considering both seismic load and groundwater are mainly including analytical method, finite element method (FEM) and laboratory test method. The finite element method and laboratory test method are most common methods for dynamic response analysis of slope [23],[24],[25],[26]. Griffiths [27] made comparison between the finite element method and other solution methods, and found that the finite element method is a more powerful alternative to the traditional pseudo-static method. It could be noticed that the finite element method is a feasible method to study the dynamic response and failure mode of the slope considering the coupling effect of seismic load and groundwater. Thus, our research group has conducted a study about the dynamic response and failure mode of the sandy slope which is affected by the coupling of groundwater and seismic load based on finite element method and laboratory test method.

## Materials and Methods

We first build the numerical simulation model to evaluate the dynamic response and failure mode of the slope under the coupling of groundwater and seismic load. Then the seismic waves are selected as the input loads. Finally, the shaking table test is conducted to evaluate the accuracy of our proposed numerical simulation model of the slope.

### Finite element model of the slope

In this section, we build the numerical simulation model to evaluate the dynamic response and failure mode of the slope under the coupling of groundwater and seismic load. A slope of Zhunshuo railway CKl62 + 075 ~ CKl63 + 075 in China's Shanxi Province is used as the research object (S1 Fig). The soil types of the slope o are mainly made up of sandy soil. The groundwater levels which vary with the season are commonly 2.0m above. In addition, the length, height and angle of the slope are 17 m, 12m and 35° respectively.

The numerical simulation model of the slope is built by a finite element software PLAXIS, as depicted in S2 Fig. The total length of the model is 62m, and the height is 24m. In order to guarantee the accuracy of the calculation, the maximum size of the grid is less than 1/10 ~ 1/8 of the shortest wave length of the input seismic waves. Mohr-Coulomb elasto-plastic model is used to model the stress-strain behavior of the soil. And the grid size of the numerical simulation model is 0.5m (S2 Fig). Furthermore, the left, right and bottom boundary are set as viscoelastic artificial boundaries.

In the numerical simulation model, the quality damping coefficient *α* and the stiffness damping coefficient *β* are fixed as 0.2 and 0.0019 respectively. So the damping coefficients of the numerical simulation model are calculated by Rayleigh damping formula, as illustrated in Eq (1).

[C]=α[M]+β[K] (1)

where *α* denotes the quality damping coefficient, and *β* is the stiffness damping coefficient.

Moreover, the quality damping coefficient and the stiffness damping coefficient are computed by Eqs (2) and (3).

$$\alpha =\frac{2\omega_{i}\omega_{j}\left(\xi_{i}\omega_{j}-\xi_{j}\omega_{i}\right)}{\omega_{j}^{2}-\omega_{i}^{2}}$$ (2)

$$\beta =\frac{2\left(\xi_{i}\omega_{j}-\xi_{j}\omega_{i}\right)}{\omega_{j}^{2}-\omega_{i}^{2}}$$ (3)

where *ω*_*i*_ represents the natural frequency of the first model, *ω*_*j*_ corresponds to the natural frequency of the second model and the range of the conventional damping ratios *ξ*_*i*_, and *ξ*_*j*_ are 2%~7%.

To provide a reference for making the slope test model, screening experiment is carried out and the grain size distribution is obtained (S3 Fig).

Based on the indoor experiment, the physical parameters of the slope are shown in Table 1.

**Table 1:** Physical parameters of slope.

Soil	Poisson's ratio	Elastic Modulus /Mpa	Gravity /KN·m^-3^	Cohesion /KPa	Friction angle /°	Saturated water content /%	Coefficient of permeability /cm·s^-1^
Sandy soil	0.3	50.4	17.5	11.42	35.23	30	5e-5

### Selection of the seismic waves

The basic intensity of the region of the slope is 7 degrees, and the site is II. According to *CODE FOR SEISMIC DESIGN OF RAILWAY ENGINEERING (GB50111-2006)*[28] of China, the basic acceleration value of severe earthquake is 0.21g, as shown in Table 2. And the peak acceleration of different seismic waves is adjusted for 0.21g.

**Table 2:** Basic earthquake acceleration value.

Earthquake category	6 degrees	7 degrees	8 degrees	9 degrees
Frequent earthquake	0.02g	0.04g	0.07g	0.14g
Rare earthquake	0.11g	0.21g	0.38g	0.64g

Refer to Japanese seismic code, the far field seismic waves (Type I: T1-II-1 and T1-II-3) and the near field seismic waves (Type II: T2-II-1 and T2-II-3) are used, as shown in Table 3. The acceleration time histories of the four seismic waves are shown in S4 Fig. Because the failure of the slope is mainly affected by the horizontal earthquake, the influence laws of horizontal earthquakes on the slope are only considered.

**Table 3:** Characteristics of seismic waves.

Seismic waves	Names	Magnitude	Distance to epicenter/km	Record location
T1-II-1	Hyūganada earthquake(1968)	7.5	100	Foundation of Itajima bridge
T1-II-3	East off Hokkaido earthquake (1994)	8.1	178	Onnetō bridge
T2-II-1	Kobe (1995)	7.2	16	JR Takatori station
T2-II-3	Kobe (1995)	7.2	11	Osaka gas supply Institute

Seismic response spectrums of different seismic waves are obtained by Fourier transform (S5 Fig).

The response spectrums are obtained when the damping is 5% (S5 Fig). Predominant periods of the far field seismic waves are mainly distributed in 0.25~1.5s. The acceleration values decrease slowly with the increasing of natural vibration period. The predominant periods of the near field seismic waves are distributed in 0~0.8s. The acceleration values decrease faster than that of far field seismic waves with the increasing of natural vibration period.

## Shaking Table Test of the Sandy Slope

The test is to research the permanent displacements of the slope in groundwater level 0m, 0.4m, 0.6m and 1.2m. The size of vibration table and test model is 1.5m×1.5m and 1.96 m×0.96m×1.2m respectively. In addition, the height of the model is 1.4m and the slope rate is 1:1.5. The Physical quantity of the model are calculated by law of similarity, as shown in Table 4. To keep the sandy soil uniform, the sandy slope is stirred repeatedly. The sponge whose thickness is 20mm is to reduce the reflection of seismic waves at the border of the slope. The test model is shown in S6 Fig.

**Table 4:** Similarity constants.

Physical quantity	Similarity relation	Similarity constants
Dynamic shear strength	*C*_|*τ*|_	4.85
Length *L*	*C*_L_	12
Density *ρ*	*C*_*p*_	1
Acceleration a	$C_{a}=C_{\left|\tau \right|}C_{\rho }{}^{-2/n}C_{L}{}^{-1}$	0.40
Time *T*	$C_{T}=C_{K}{}^{-\frac{1}{2}}C_{\rho }{}^{-\frac{1}{2n}}C_{L}{}^{\frac{2n-1}{2n}}$	6.45
Strain $\gamma /{\bar{\gamma }}^{}$	$C_{\gamma /\bar{\gamma }}$	1
Dynamic displacement *u*	$C_{u}=C_{\left|\tau \right|}C_{K}{}^{-1}C_{\rho }{}^{-\frac{1}{n}}C_{L}{}^{\frac{n-1}{n}}$	16.80
Dynamic shear modulus *C*_*G*max_	$C_{G\text{max}}=C_{K}C_{\rho }{}^{\frac{1}{n}}C_{L}{}^{\frac{1}{n}}$	3.46
Frequence *ω*	$C_{w}=C_{K}{}^{-\frac{1}{2}}C_{\rho }{}^{-\frac{1}{2n}}C_{L}{}^{-\frac{2n-1}{2n}}$	0.16
Damping Ratio	*C*_*λ*_ = 1	1

Test is done on the hydraulic one-way shaking table (ES-15/KE-2000). The main technical indicators of the shaking table test are as follows:

The maximum test load is 5000kg. The maximum acceleration is 20m/s^2^. The rated speed is 0.5m/s. Equipment and monitoring system are shown in S7 Fig.

### Simulation of the underground water level and sensor layout

Simulation of underground water level determines the accuracy of test results. Test program of the simulation of underground water level is shown in S8 Fig.

As shown in S8 Fig, the water is injected at the left side of the slope, and groundwater levels are controlled at the height of 0.4m, 0.6m and 1.2m. The height of the underground water level on the right side is always controlled at the height of the slope toe by turning on the tap. The stable seepage field inside the slope will be formed after a long time seepage of water.

In order to obtain the accurate data of the test, many sensors are deployed along the height of the slope. The layout of monitoring sensors of the test model is shown in S9 Fig.

### Natural frequency analysis of the model

The first order natural frequency is obtained by frequency sweep test in different underground water levels 0m, 0.4m, 0.8m and 1.2m, as shown in Table 5. And S10 Fig lists spectrograms of the groundwater levels 0m and 0.8m.

**Table 5:** Test results of the frequencies.

Condition	Natural frequency /Hz	Period/s
0m	29.23	0.034
0.4m	17.54	0.057
0.8m	12.65	0.079
1.2m	10.20	0.098

As shown in Table 5 and S10 Fig, the natural vibration period increases gradually with the increasing of groundwater levels. And the natural frequency decreases from 29.23Hz in the groundwater level 0m to 10.2Hz in the groundwater level 1.2m. The period in the groundwater level 1.2m increases 2.88 times than that in the groundwater level 0m. Thus, the existence of the groundwater has a great effect on dynamic characteristics of the slope.

## Results and Discussion

### Pseudo-static analysis of the slope in different groundwater levels

The safety factors of the slope in groundwater levels 0m, 12m, 14m, 16m, 18m, 20m, 22m and 24m are calculated, as described in S11 Fig.

As shown in S11 Fig, safety factors decrease when the groundwater levels increase, and the safety factors dramatically decrease when the groundwater levels reach a critical depth. Therefore, the slope should be strengthened to guarantee the stability in the high groundwater level. If not, it easily occurs collapse or landslides in the high groundwater level.

The displacement nephograms in different groundwater levels are obtained (S12 Fig).

As shown in S12 Fig, the maximum displacement in no groundwater is located at the middle-upper of the slope. The horizontal displacement increases when the groundwater level increases. When the groundwater level reaches 22 m, the horizontal peak displacement increases by 58.3% than that in no groundwater. In addition, the maximum deformation gradually shifts from the slope top to the slope toe with the increasing of the groundwater level. And the depth of the slip plane extends to the inside of the slope. The effective stress of the slope toe drops with the increasing of groundwater level, which makes the displacement increase until the slope sliding.

### Effect of different ground water level on dynamic response of the slope

In order to evaluate the seismic dynamic responses in different underground water levels, the height of the water head (H) are defined as 12, 14, 16, 18, 20, 22 and 24m. The horizontal peak acceleration values in different underground water levels along the height of the slope are obtained (S13 Fig).

It can be seen from S13 Fig, the peak acceleration of the slope decreases with the increasing of groundwater level when the slope is under near and far field earthquakes, which shows that the groundwater has the role of reducing vibration. The peak accelerations along the height of the slope show different variation trends when the slope is under near and far field earthquakes. The horizontal peak accelerations tend to increase along the height of the slope in different groundwater levels when the slope is under far field earthquakes (Figure A and Figure B in S13 Fig). The acceleration appears the maximum value at the slope top, and the slope has remarkable whiplash effect. Peak accelerations along the height of the slope are greater than the peak accelerations of the input seismic waves, which shows that the sandy slope has the amplification effect to the seismic waves. The peak accelerations decrease first and then increase along the height of the slope in different groundwater levels when the slope is under near field earthquakes (Figure C and Figure D in S13 Fig). The minimum value appears at the slope middle, while the maximum value appears at the slope top when the slope is under near field earthquakes. The reasons for different rules of accelerations under different type earthquakes are mainly that the seismic wave will produce the splitting phenomenon, meanwhile the seismic wave decomposes into the reflection wave and converted wave. And the superimposition of the various waves forms complex seismic wave field, which makes the acceleration increase sharply at the slope top.

Time histories of the accelerations (T1-II-1 and T2-II-1) at the slope toe, middle and top in groundwater levels 0m and 24m respectively are obtained, as illustrated in S14 and S15 Figs.

As shown in S14 and S15 Figs, the existence of the soil material damping could absorb some parts of the wave energy. And there is filtering function to high frequency seismic waves. The phenomenon of the filtering function to high frequency seismic waves is more obvious in high groundwater level. When the seismic wave spreads across the soil, its frequency spectrum characteristics have changed significantly. Acceleration values (1~2.5 Hz) increase when acceleration values (2.5~10Hz) decrease from the slope top to the slope toe.

Displacement response could provide an important reference for slope stability evaluation when the slope is under earthquakes. Thus, the change laws of peak displacement along the slope height in different groundwater levels are obtained, as shown in S16 Fig.

As shown in S16 Fig, the horizontal peak displacements in groundwater are greater than that in no groundwater when the slope is under near and far field earthquakes. And the horizontal peak displacements under far field earthquakes are greater than that under near field earthquakes. The horizontal peak displacement increases with the increasing of groundwater levels, and decreases with the slope height increasing. The effect of far field earthquake on the deformation of slope toe is greater, while the effect of near-field earthquake on the deformation of the slope top is greater. Therefore, the slope top and toe should be protected as the key position when the slope is under earthquakes. In a word, the existence of the groundwater has a greater effect on horizontal displacement of the slope, and the effect is more obvious when the slope is under the far field earthquake.

Time histories of displacements (T1-II-1 and T2-II-1) at the slope toe, middle and top in groundwater levels 0m and 24m are calculated, as shown in S17 and S18 Figs.

As shown in S17 and S18 Figs, the peak displacement under different earthquakes appears in different time. The slope occurs plastic deformation with the increasing of groundwater levels. The slope deformation varies hugely at the initial moment when the slope is under near field earthquake T2-II-1. There is an obvious accumulation of the displacements at the time 8s and 9s, which means that the slope produces a larger plastic deformation. The slope deformation varies little at the initial moment when the slope is under far field earthquake T1-II-1, and there is a significant accumulation of the slope displacement at the time16s and17s.

### Dynamic pore water pressure in different groundwater levels

Values of dynamic pore water pressure in different underground waters are obtained, as shown in S19, S20, S21 and S2 Figs.

As shown in S19, S20, S21 and S2 Figs, the values of dynamic pore water pressure rise sharply within a short time when the slope is under near field earthquakes and far field earthquakes. The dynamic pore water pressure is too late to dissipate, and it shows a large fluctuations. The value of dynamic pore water pressure at the slope toe is greatest, and the slope toe is also the position occurring easily shear failure when the slope is affected by the dynamic pore water pressure. Thus, the toe of the slope should be as the key position protected in the actual engineering.

### Effect of groundwater on seismic failure mode of the slope

The effect of groundwater on the failure mode of the sandy slope which is under the earthquake T1-II-1 is evaluated, and meanwhile we fix the peak acceleration to 4m/s^2^. The destroying process is recorded by the video, and some pictures are listed, as depicted in S23 and S24 Figs.

As shown in S23 and S24 Figs, the destruction of the slope first appears at the top position when there is no groundwater, and the whipping effect of the slope is very evident. The destruction of the slope first appears at the toe position when the underground water level is 0.8m. And the slope toe cracks when the slope is under earthquake. As the dynamic pore water pressure continues to rise, soil liquefaction appears at the slope toe and the slope finally is destroyed. In addition, there is a significant subsidence phenomenon when the slope is after the earthquake.

## Comparison Analysis of Results of the Indoor Test and that of the Finite Element Method

Results of the shaking table test about the pore water pressure, the acceleration response and the displacement response have been presented in previous manuscript [29]. However, the main purpose of this experiment is to make comparison of our results and results of the finite element method. The test results of the indoor model are translated into corresponding values of the slope prototype by the analogous theory. And these corresponding values of the slope prototype will be compared with results of the finite element method. The calculation results are analyzed when the slope is under earthquakes T1-II-1 and T2-II-1 and meanwhile the groundwater levels are 18m a 22m, as illustrated in S25 Fig. Moreover, the differences of horizontal peak displacement values between results of the indoor test and that of the finite element method are compared, as shown in S26 Fig.

As shown in S25 and S26 Figs, the test values of the peak displacements and accelerations are greater than results of the finite element method. However, the maximum deviation between results of the indoor test and results of the finite element method is less than 10%, which shows the accuracy of results of the finite element method.

The change laws of dynamic pore water pressure values at the slope toe when the underground water levels are 14m and 22m are analyzed, as shown in S27 and S28 Figs.

It can be seen in S27 and S28 Figs, values of the dynamic pore water pressure measured by the indoor test are less than values of the finite element for almost all the groundwater levels. However, the fluctuations laws of the indoor test and the finite element method are consistent. In addition, the maximum error between values of the indoor test and that of the finite element method is less than 15%.

## Conclusions

The main research conclusions are as follows:

1. The safety factor will dramatically decrease when the groundwater level reaches a critical depth. And the maximum deformation appears at the middle-upper of the slope when the slope is in no groundwater, while the maximum deformation gradually shifts from the slope top to the slope toe with the increasing of the groundwater level. Furthermore, the depth of the slip plane extends to the inside of the slope.
2. The peak accelerations of the slope decrease with the increasing of underground water level when the slope is under near and far field earthquakes, which shows that the existence of the groundwater has the role of reducing vibration. However, the peak accelerations along the height of the slope show different variation trends when the slope is under the near and far field earthquakes. The maximum values of the accelerations appear at the slope top when the slope is under far and near field earthquakes.
3. Far-field earthquake affects mainly the deformation of the slope toe, while near-field earthquake has more influence on the deformation of the slope top. Therefore, the top and toe of the slope should be protected as the key position when the slope is under different earthquakes. The existence of the groundwater has a greater effect on horizontal displacement of the slope, and especially the influence of the groundwater on the slope under far field earthquake is more obvious.
4. Natural vibration period of the slope increases gradually with the increasing of the groundwater levels. The destruction of the slope first appears at the top position when there is no groundwater, and the whipping effect of the slope is very evident. The destruction of the slope occurs first at the toe position when there is groundwater. Meanwhile, the top of the slope has the settlement phenomenon when the earthquake is end.

## Supporting Information

S1 Fig Engineering field figure. (TIF)

S2 Fig The calculation model of the slope. (TIF)

S3 Fig Grain size distribution. (TIF)

S4 Fig Seismic waves. T1-II-1 (Figure A in S4 Fig). T1-II-3 (Figure B in S4 Fig). T2-II-1 (Figure C in S4 Fig). T2-II-3 (Figure A in S4 Fig).
(TIFF)

S5 Fig Seismic response spectrum. (TIF)

S6 Fig The test model of the slope. (TIF)

S7 Fig Vibration equipment. (TIF)

S8 Fig Simulation of the groundwater levels. (TIF)

S9 Fig Layout of monitoring sensors. (TIF)

S10 Fig The spectrograms. 0m (Figure A in S10 Fig). 0.8m (Figure B in S10 Fig).
(TIF)

S11 Fig The safety factors of the slope with underground water level. (TIF)

S12 Fig The displacement nephograms in different groundwater levels. 0m (Figure A in S12 Fig). 14m (Figure B in S12 Fig). 18m (Figure C in S12 Fig). 22m (Figure D in S12 Fig).
(TIFF)

S13 Fig Effects of different groundwater levels on horizontal peak accelerations. T1-II-1 (Figure A in S13 Fig). T1-II-3 (Figure B in S13 Fig). T2-II-1 (Figure C in S13 Fig). T2-II-3 (Figure D in S13 Fig).
(TIFF)

S14 Fig Acceleration response and spectrum under T1-II-1. 0m (Figure A in S14 Fig). 24m (Figure B in S14 Fig).
(TIFF)

S15 Fig Acceleration response and spectrum under T2-II-1. 0m (Figure A in S15 Fig). 24m (Figure B in S15 Fig).
(TIFF)

S16 Fig Effects of different groundwater levels on horizontal peak displacement. T1-II-1 (Figure A in S16 Fig). T1-II-3 (Figure B in S16 Fig). T2-II-1 (Figure C in S16 Fig). T2-II-3 (Figure D in S16 Fig).
(TIFF)

S17 Fig Displacements response under T1-II-1. 0m (Figure A in S17 Fig). 24m (Figure B in S17 Fig).
(TIFF)

S18 Fig Displacements response under T2-II-1. 0m (Figure A in S18 Fig). 24m (Figure B in S18 Fig).
(TIFF)

S19 Fig Dynamic pore water pressure under T1-II-1. 12m (Figure A in S19 Fig). 24m (Figure B in S19 Fig).
(TIFF)

S20 Fig Dynamic pore water pressure under T1-II-3. 12m (Figure A in S20 Fig). 24m (Figure B in S20 Fig).
(TIFF)

S21 Fig Dynamic pore water pressure under T2-II-1. 12m (Figure A in S21 Fig). 24m (Figure B in S21 Fig).
(TIFF)

S22 Fig Dynamic pore water pressure under T2-II-3. 12m (Figure A in S22 Fig). 24m (Figure B in S22 Fig).
(TIFF)

S23 Fig Progressive failure of the slope in no groundwater. (TIF)

S24 Fig Progressive failure of the slope in groundwater level 0.8m. (TIF)

S25 Fig Comparative analysis of the peak accelerations. 18m (Figure A in S25 Fig). 22m (Figure B in S25 Fig).
(TIFF)

S26 Fig Comparative analysis of the peak displacement. 18m (Figure A in S26 Fig). 22m (Figure B in S26 Fig).
(TIFF)

S27 Fig Comparison analysis of the dynamic pore water pressure under T1-II-1. 14m (Figure A in S27 Fig). 22m (Figure B in S27 Fig).
(TIFF)

S28 Fig Comparison analysis of the dynamic pore water pressure under T2-II-1. 14m (Figure A in S28 Fig). 22m (Figure B in S28 Fig).
(TIFF)
