# Supplementary figures and images for: Effect of Different Groundwater Levels on Seismic Dynamic Response and Failure Mode of Sandy Slope
Source: PLoS One. 2015 Nov 11;10(11):e0142268. doi: 10.1371/journal.pone.0142268 (PMC4641591; doi:10.1371/journal.pone.0142268)

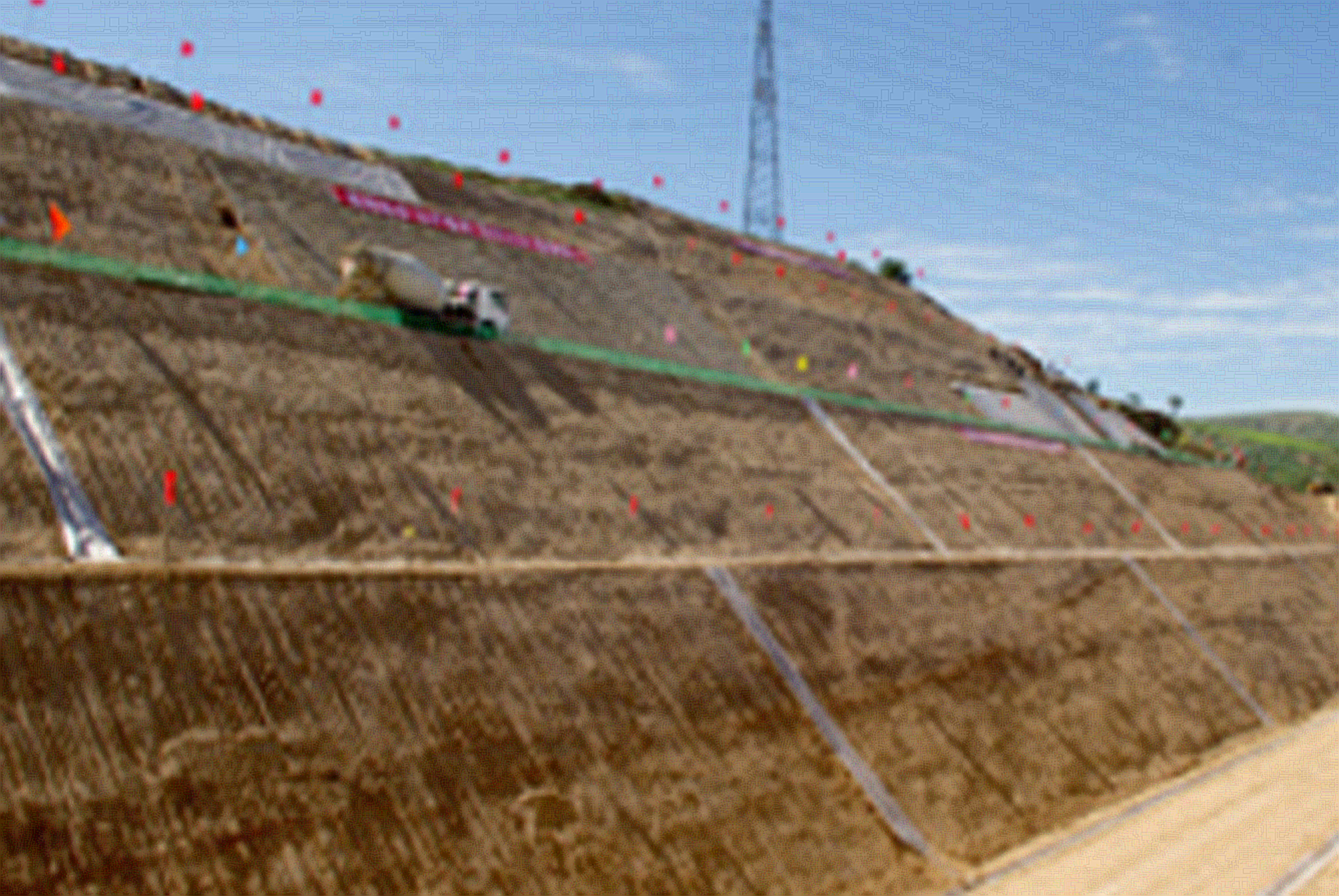

Supplement: S1 Fig — (TIF) [file pone.0142268.s001.tif]

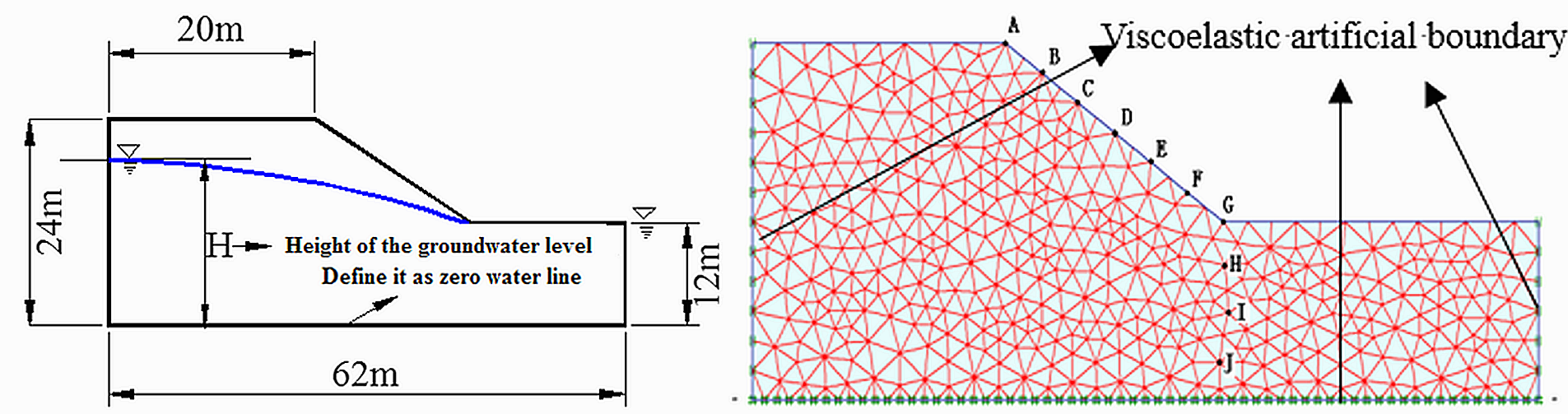

Supplement: S2 Fig — (TIF) [file pone.0142268.s002.tif]

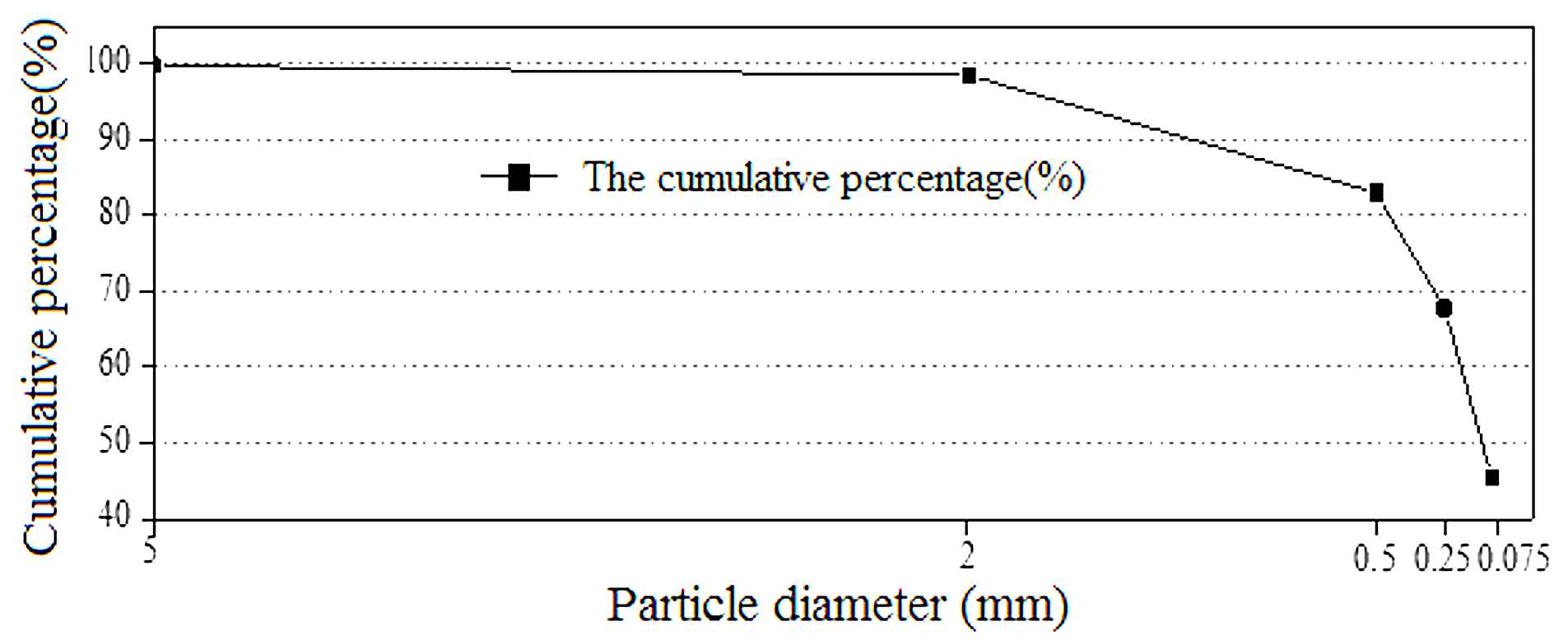

Supplement: S3 Fig — (TIF) [file pone.0142268.s003.tif]

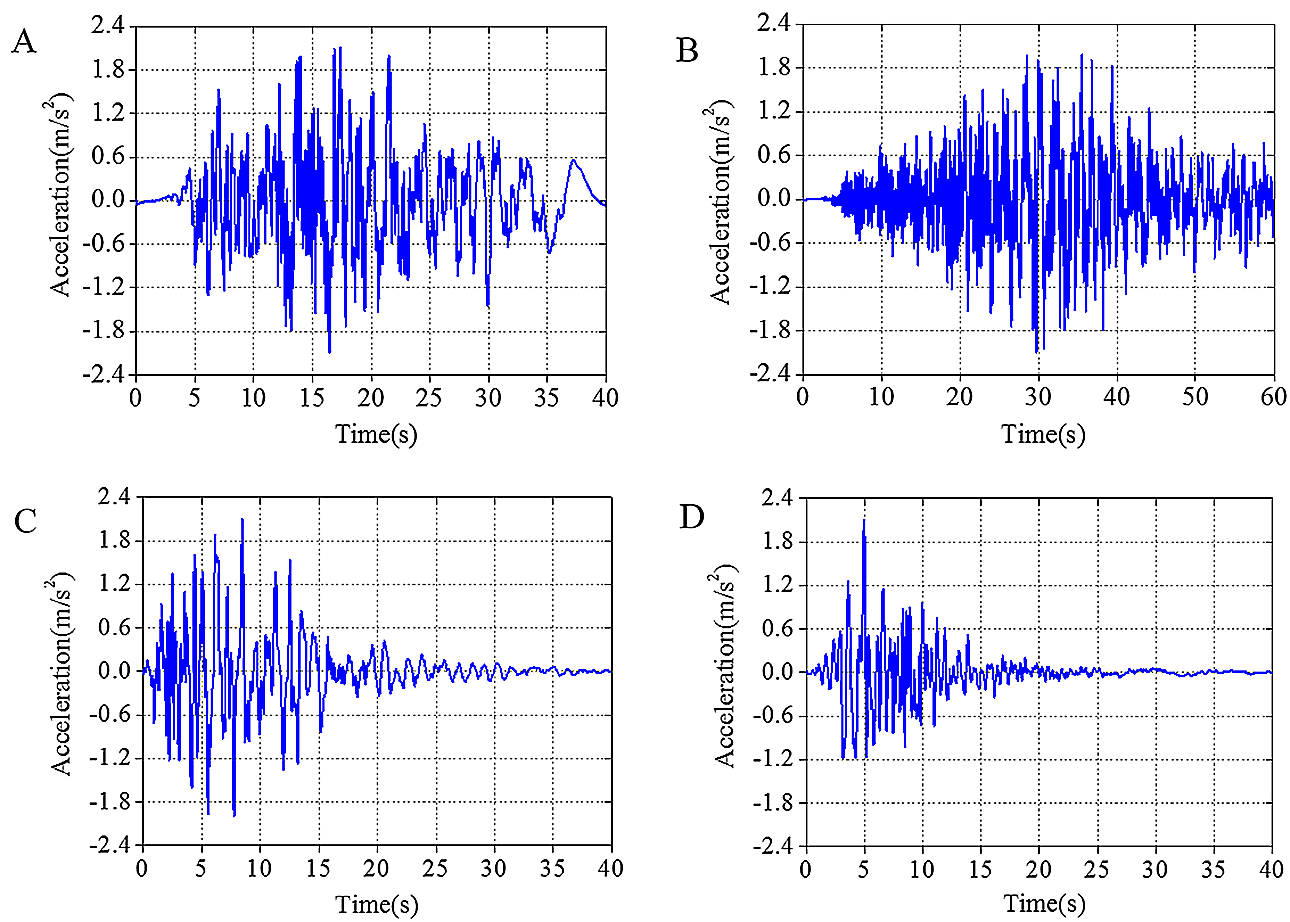

Supplement: S4 Fig — T1-II-1 (Figure A in S4 Fig). T1-II-3 (Figure B in S4 Fig). T2-II-1 (Figure C in S4 Fig). T2-II-3 (Figure A in S4 Fig). (TIFF) [file pone.0142268.s004.tiff]

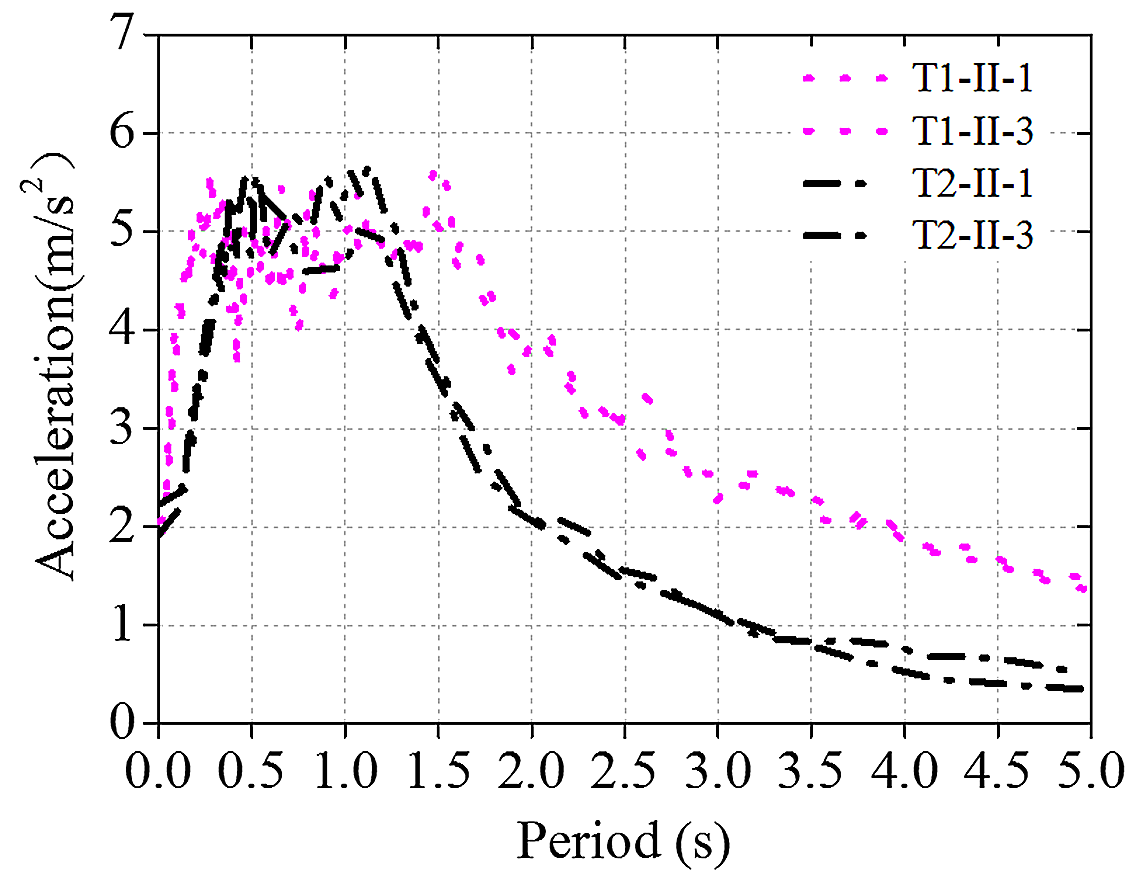

Supplement: S5 Fig — (TIF) [file pone.0142268.s005.tif]

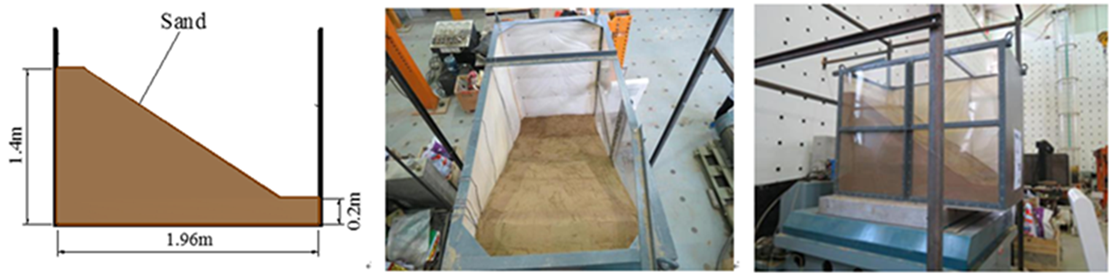

Supplement: S6 Fig — (TIF) [file pone.0142268.s006.tif]

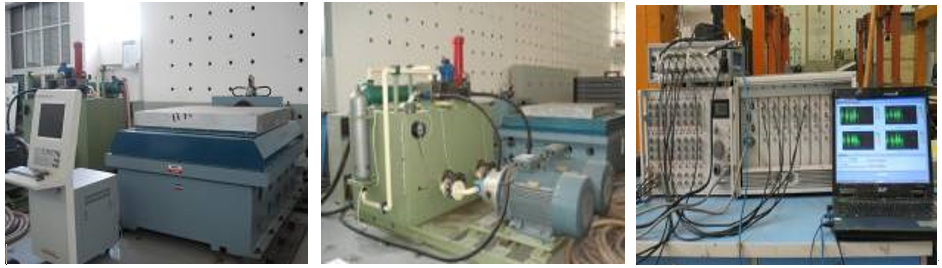

Supplement: S7 Fig — (TIF) [file pone.0142268.s007.tif]

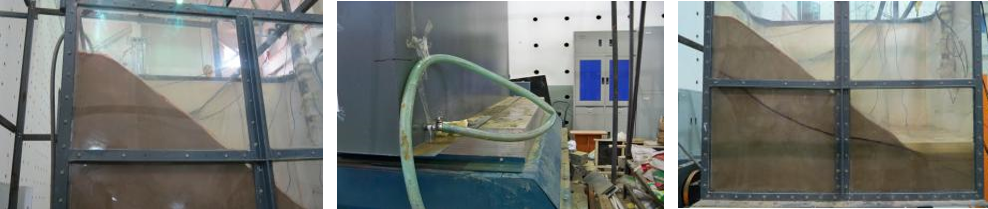

Supplement: S8 Fig — (TIF) [file pone.0142268.s008.tif]

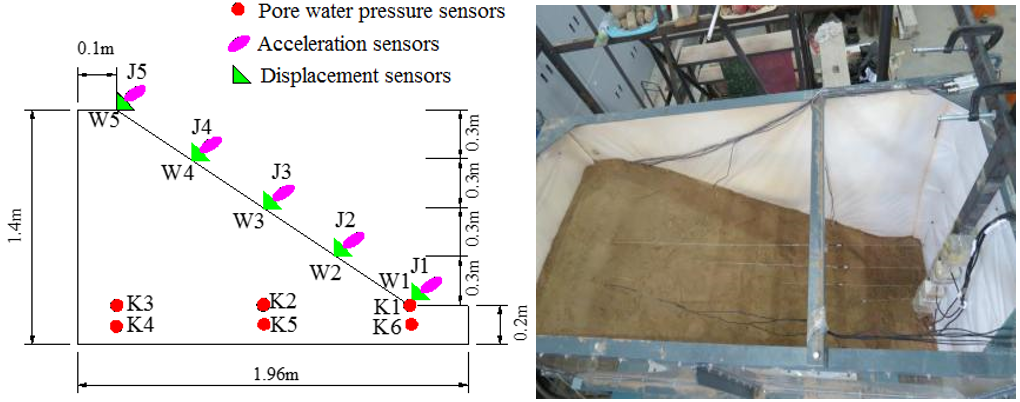

Supplement: S9 Fig — (TIF) [file pone.0142268.s009.tif]

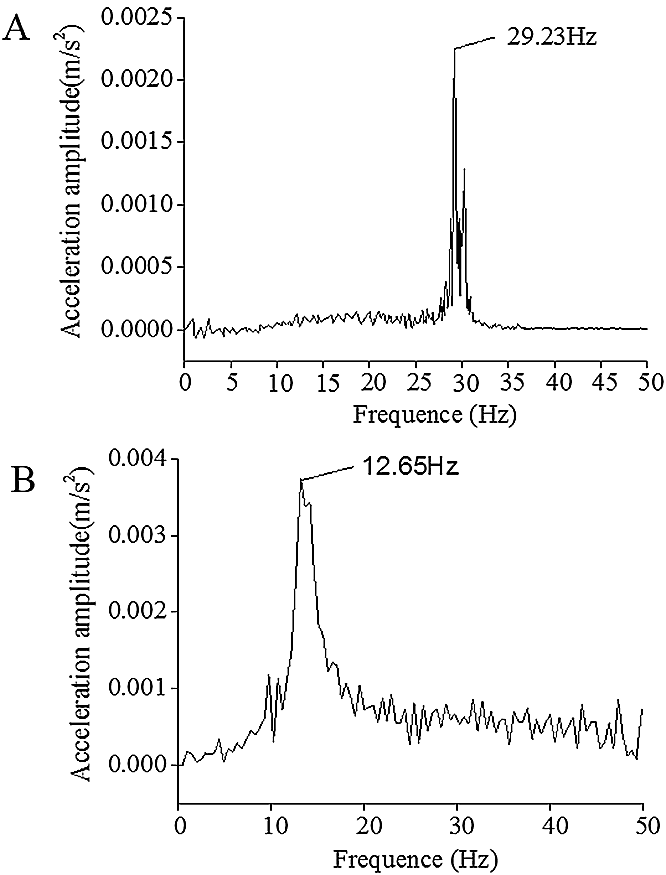

Supplement: S10 Fig — 0m (Figure A in S10 Fig). 0.8m (Figure B in S10 Fig). (TIF) [file pone.0142268.s010.tif]

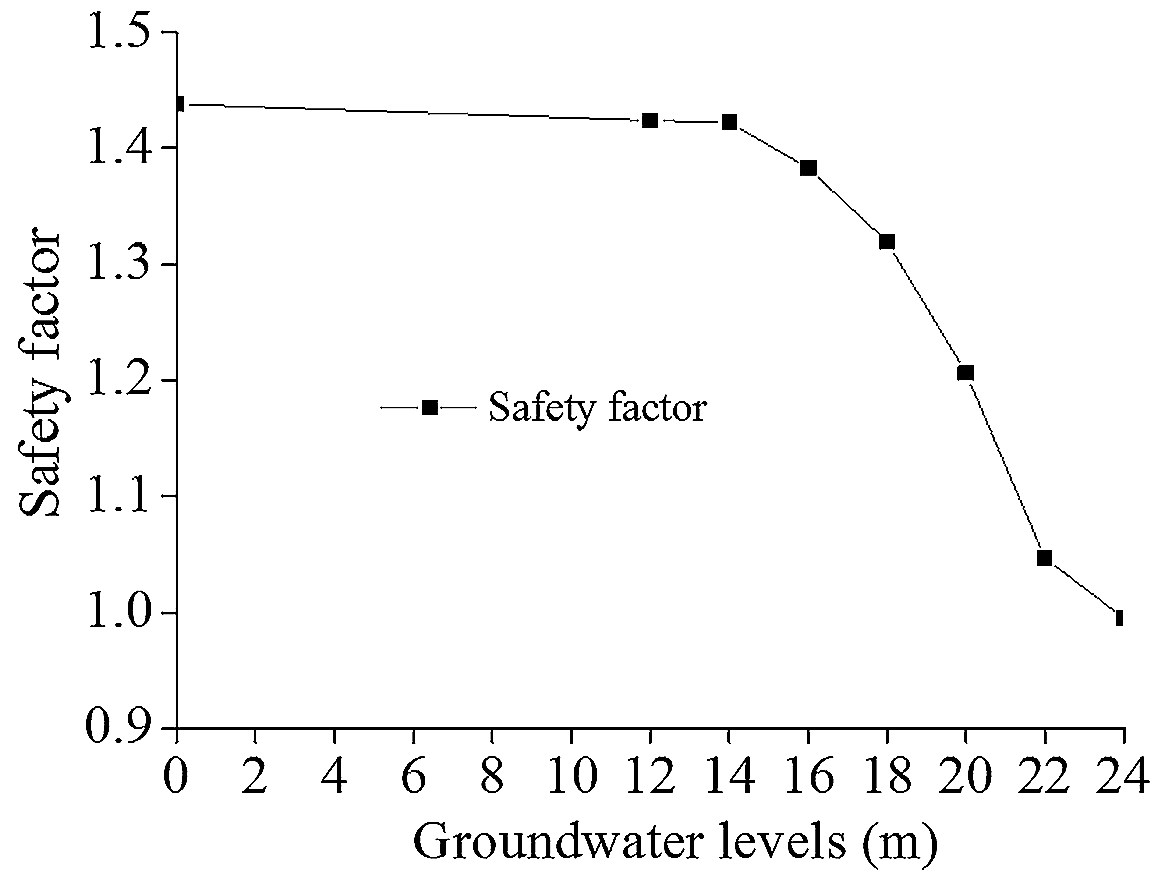

Supplement: S11 Fig — (TIF) [file pone.0142268.s011.tif]

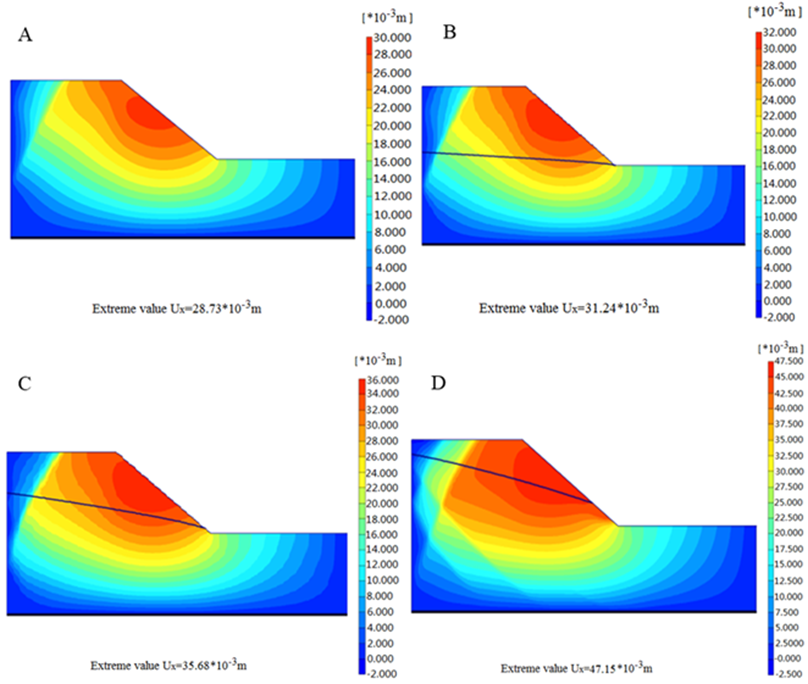

Supplement: S12 Fig — 0m (Figure A in S12 Fig). 14m (Figure B in S12 Fig). 18m (Figure C in S12 Fig). 22m (Figure D in S12 Fig). (TIFF) [file pone.0142268.s012.tiff]

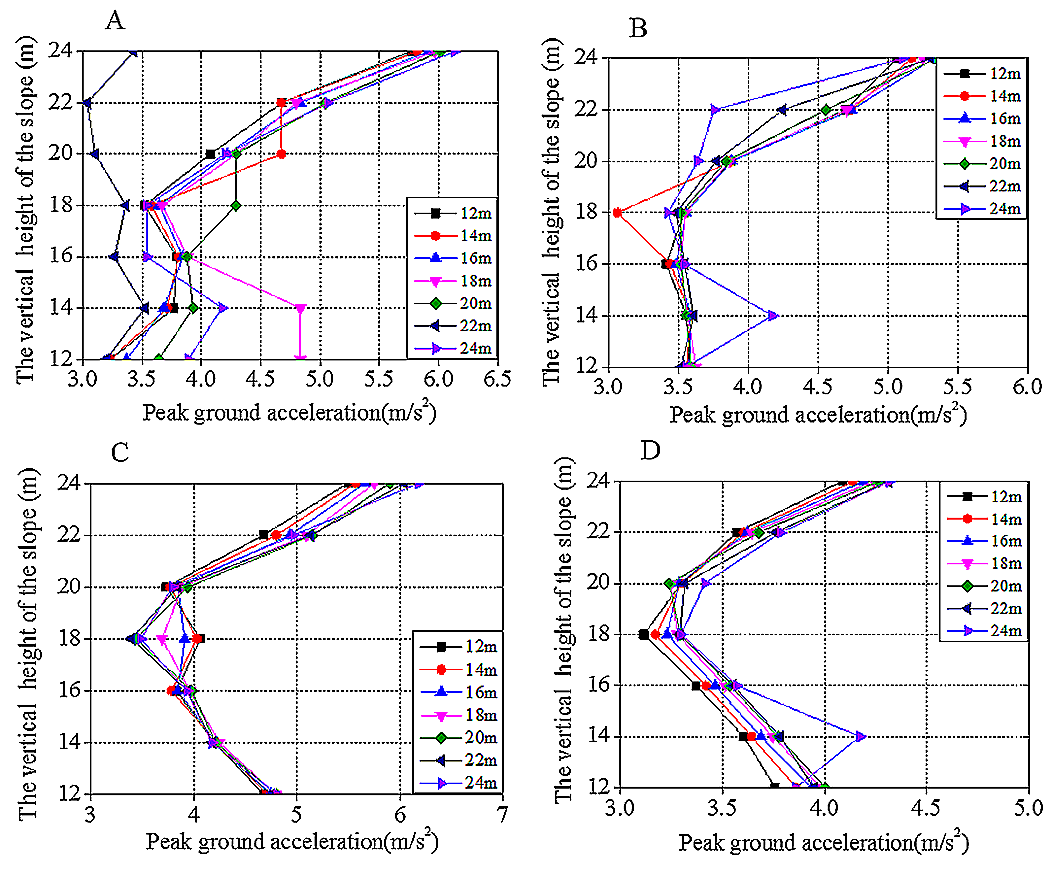

Supplement: S13 Fig — T1-II-1 (Figure A in S13 Fig). T1-II-3 (Figure B in S13 Fig). T2-II-1 (Figure C in S13 Fig). T2-II-3 (Figure D in S13 Fig). (TIFF) [file pone.0142268.s013.tiff]

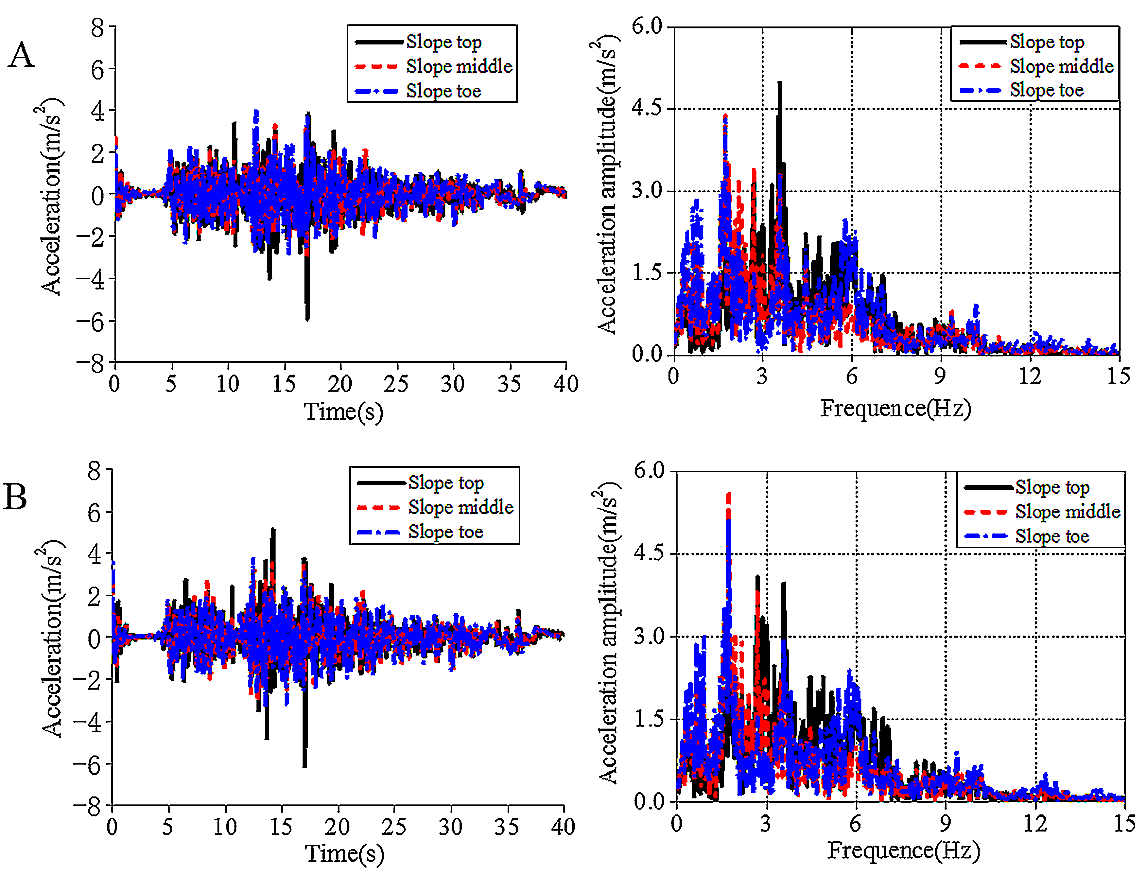

Supplement: S14 Fig — 0m (Figure A in S14 Fig). 24m (Figure B in S14 Fig). (TIFF) [file pone.0142268.s014.tiff]

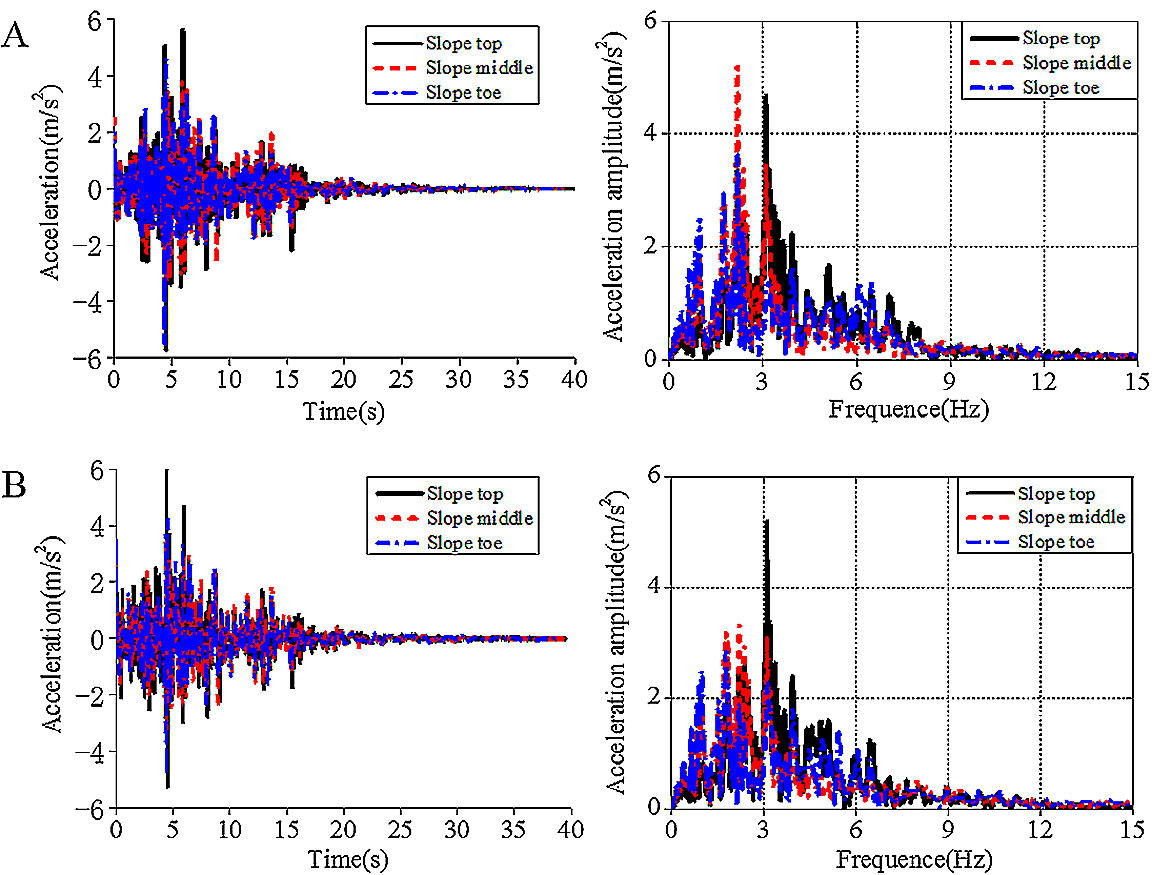

Supplement: S15 Fig — 0m (Figure A in S15 Fig). 24m (Figure B in S15 Fig). (TIFF) [file pone.0142268.s015.tiff]

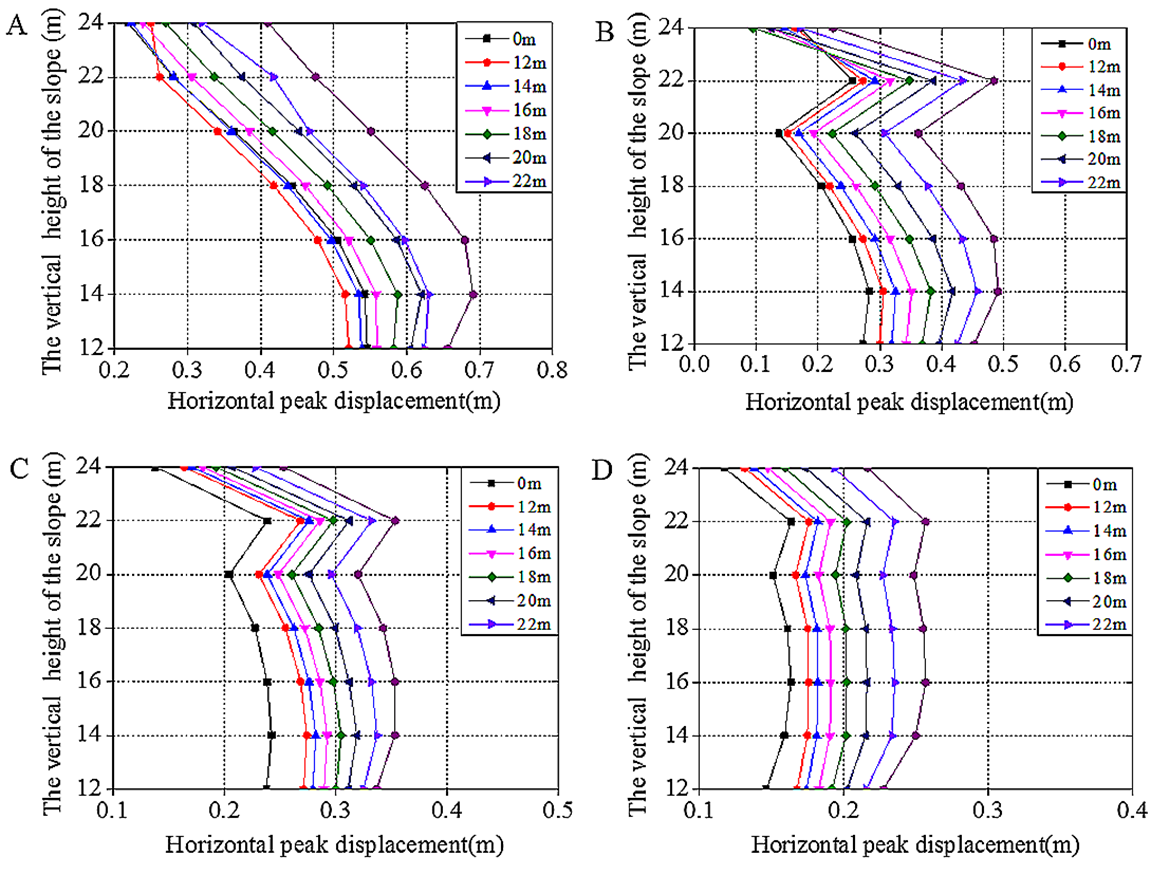

Supplement: S16 Fig — T1-II-1 (Figure A in S16 Fig). T1-II-3 (Figure B in S16 Fig). T2-II-1 (Figure C in S16 Fig). T2-II-3 (Figure D in S16 Fig). (TIFF) [file pone.0142268.s016.tiff]

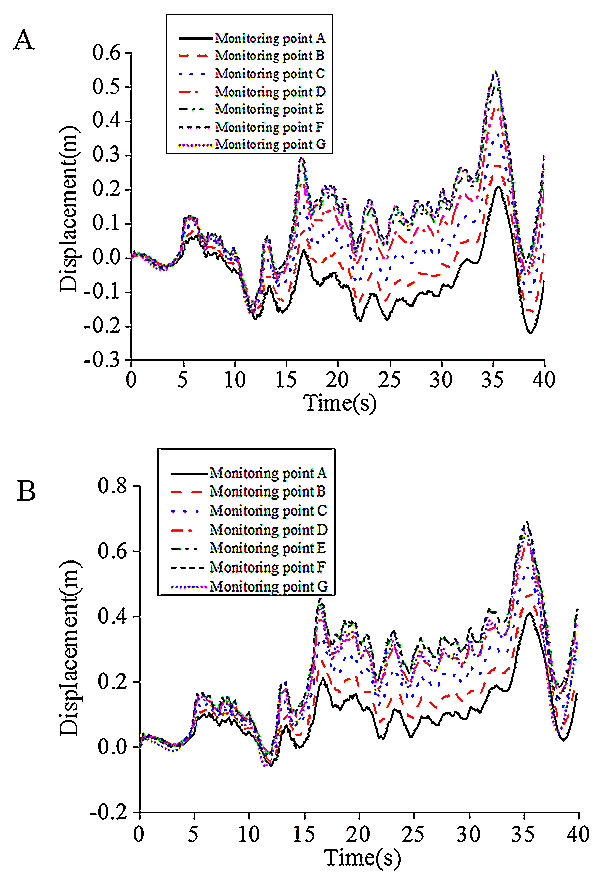

Supplement: S17 Fig — 0m (Figure A in S17 Fig). 24m (Figure B in S17 Fig). (TIFF) [file pone.0142268.s017.tiff]

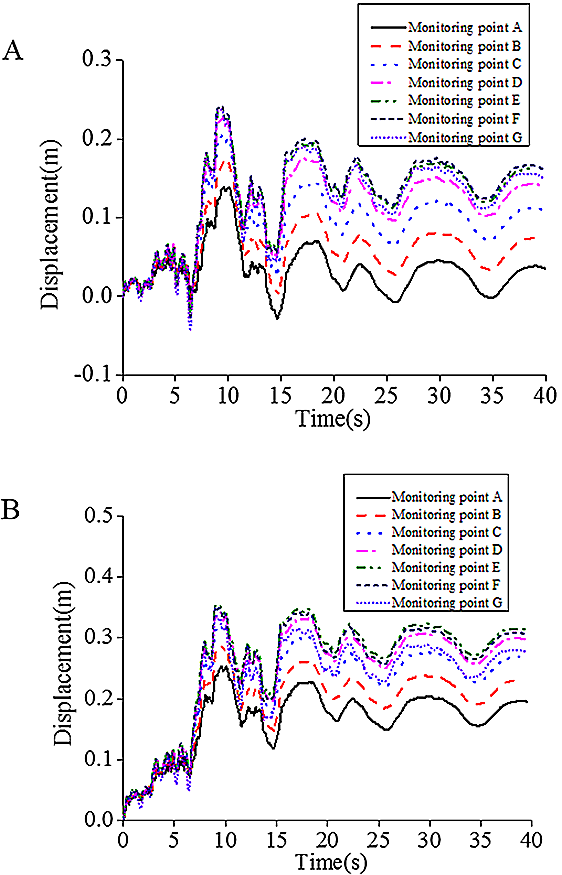

Supplement: S18 Fig — 0m (Figure A in S18 Fig). 24m (Figure B in S18 Fig). (TIFF) [file pone.0142268.s018.tiff]

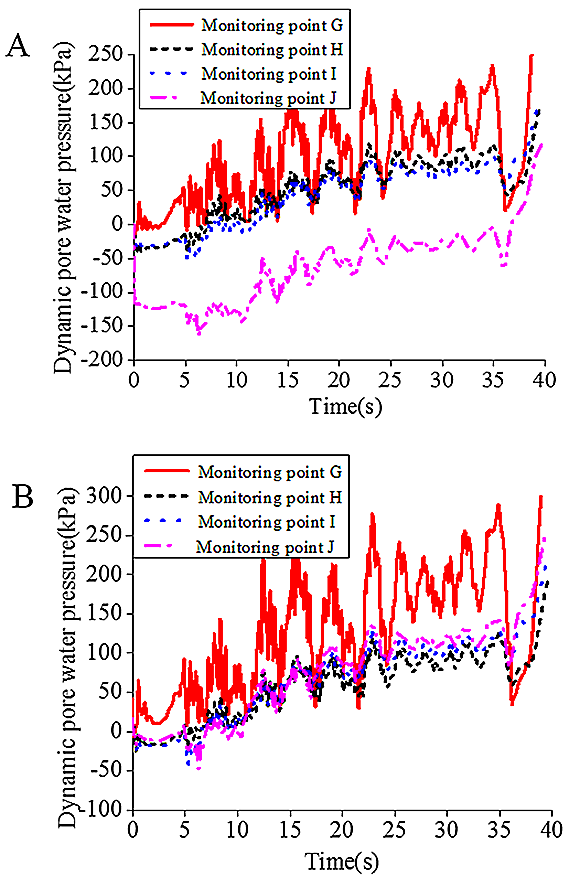

Supplement: S19 Fig — 12m (Figure A in S19 Fig). 24m (Figure B in S19 Fig). (TIFF) [file pone.0142268.s019.tiff]

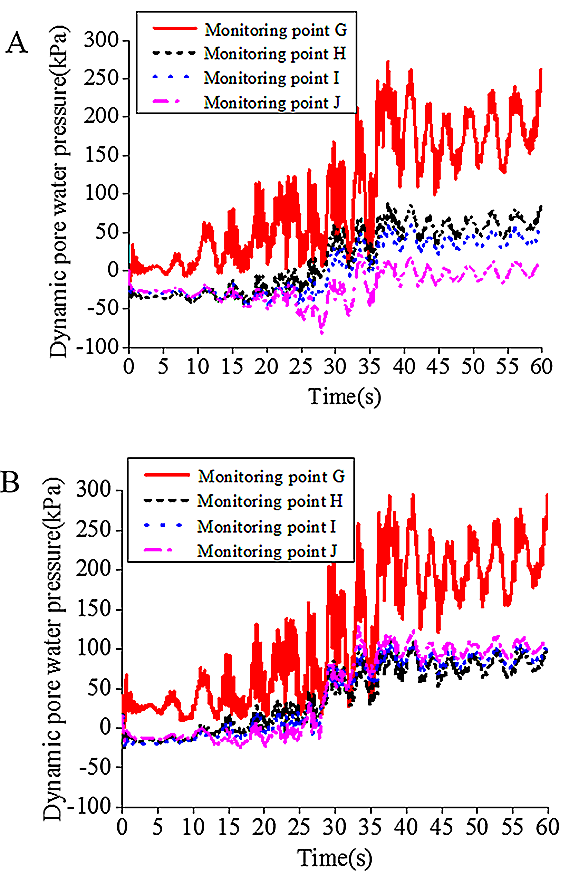

Supplement: S20 Fig — 12m (Figure A in S20 Fig). 24m (Figure B in S20 Fig). (TIFF) [file pone.0142268.s020.tiff]

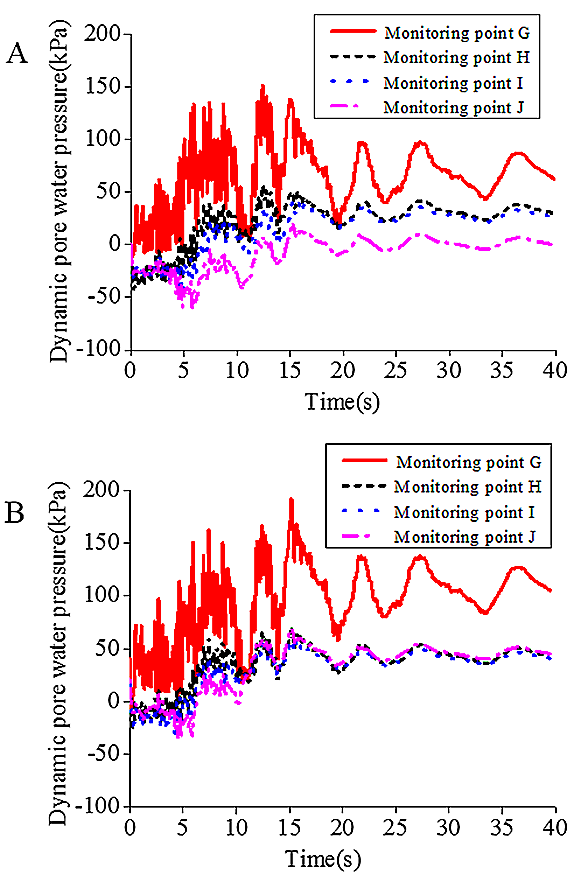

Supplement: S21 Fig — 12m (Figure A in S21 Fig). 24m (Figure B in S21 Fig). (TIFF) [file pone.0142268.s021.tiff]

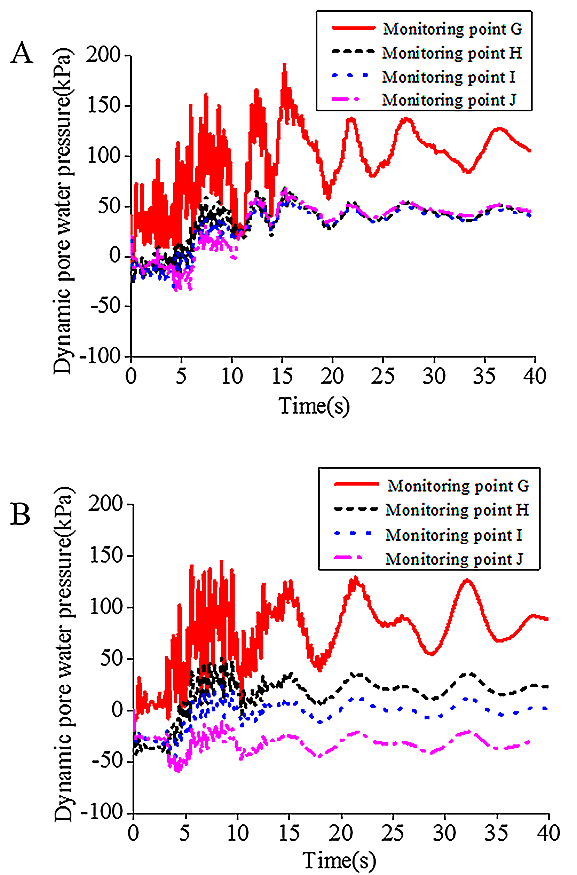

Supplement: S22 Fig — 12m (Figure A in S22 Fig). 24m (Figure B in S22 Fig). (TIFF) [file pone.0142268.s022.tiff]

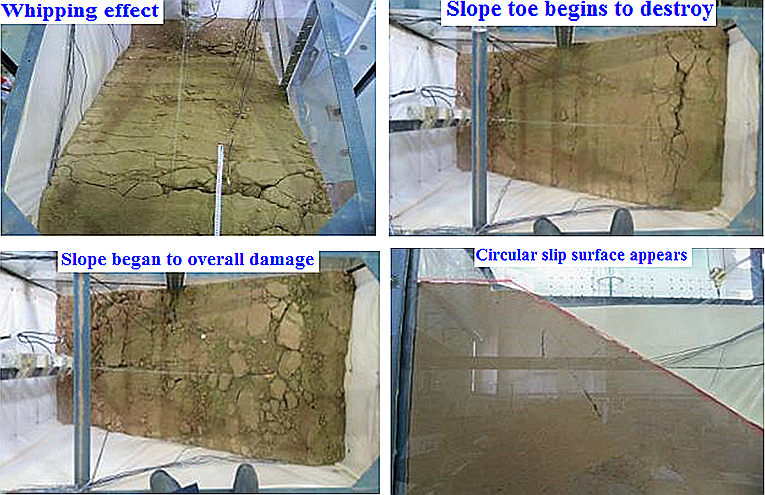

Supplement: S23 Fig — (TIF) [file pone.0142268.s023.tif]

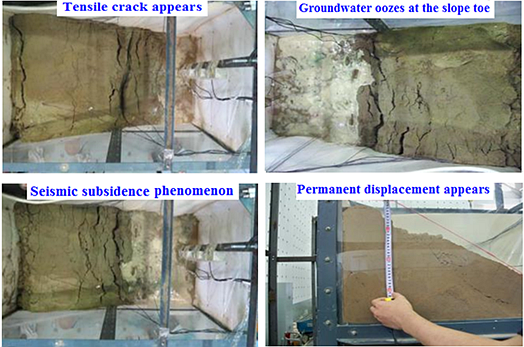

Supplement: S24 Fig — (TIF) [file pone.0142268.s024.tif]

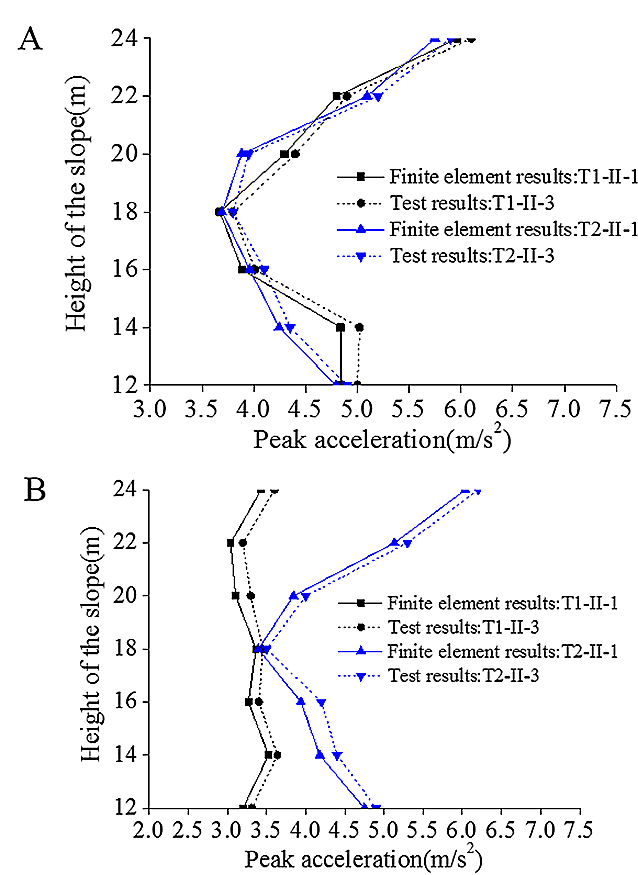

Supplement: S25 Fig — 18m (Figure A in S25 Fig). 22m (Figure B in S25 Fig). (TIFF) [file pone.0142268.s025.tiff]

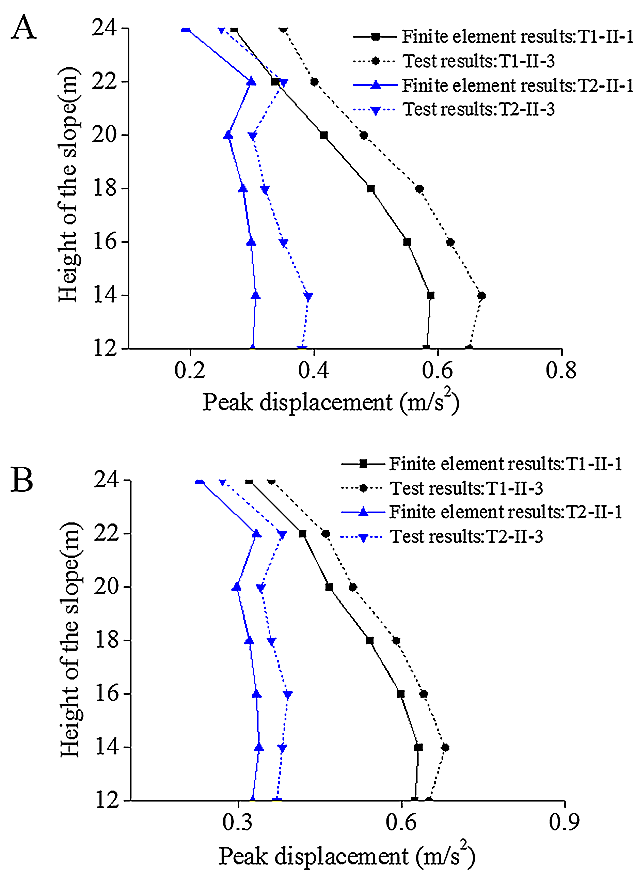

Supplement: S26 Fig — 18m (Figure A in S26 Fig). 22m (Figure B in S26 Fig). (TIFF) [file pone.0142268.s026.tiff]

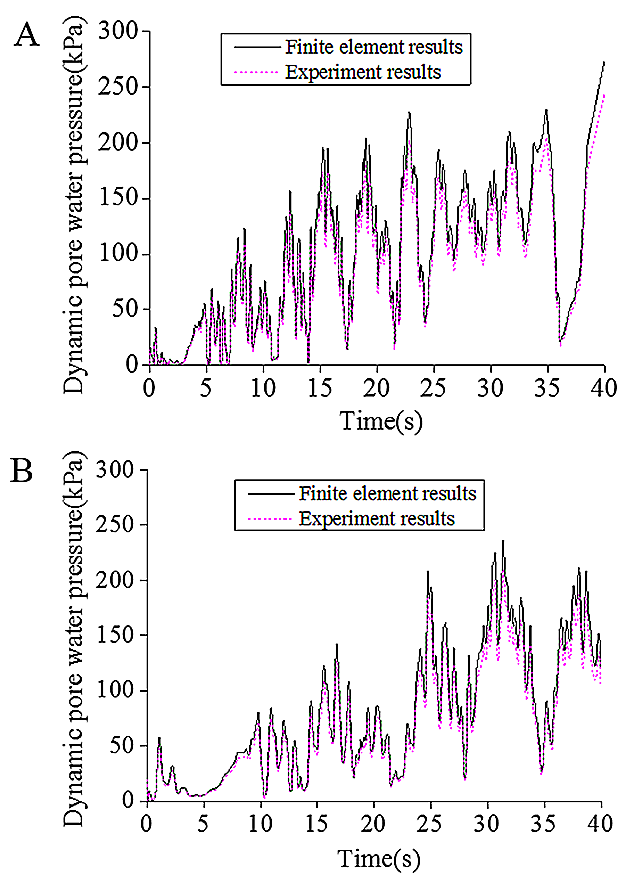

Supplement: S27 Fig — 14m (Figure A in S27 Fig). 22m (Figure B in S27 Fig). (TIFF) [file pone.0142268.s027.tiff]

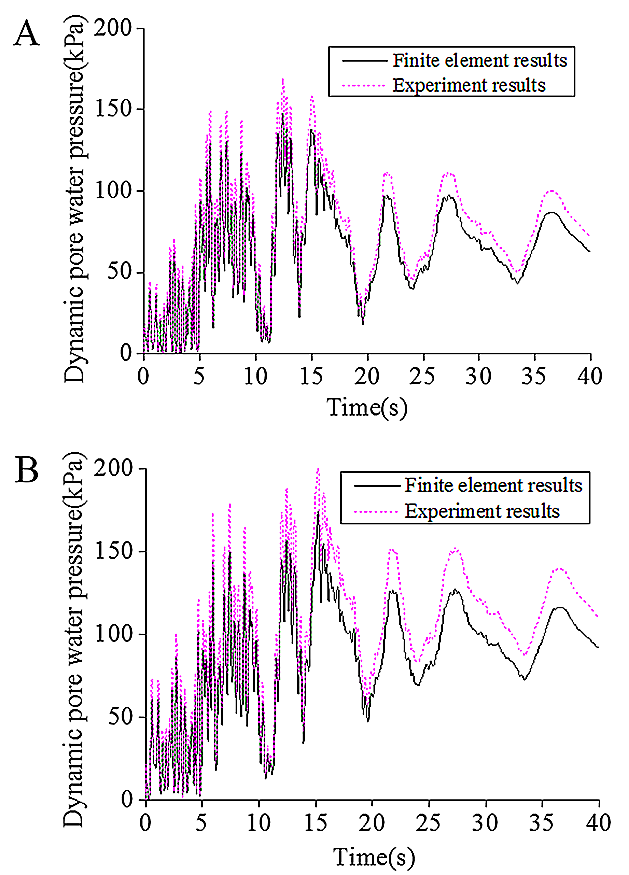

Supplement: S28 Fig — 14m (Figure A in S28 Fig). 22m (Figure B in S28 Fig). (TIFF) [file pone.0142268.s028.tiff]
